# 

**Published:** 1888-11-10

**Authors:** 


PRICE ONE PENNY.

[N]o. 111, Vol. V.—November 10th, 1888.

[Registered at the General Post Office as a Newspaper.]

Per Annum, 6s. 6d., post free.

Monthly Parts, 6d.; post free, 7½d.

Ditto per Ann., 7s. 6d. post free.

### THE HOSPITAL

A WEEKLY INSTITUTIONAL JOURNAL OF

